# Eph/ephrin signaling maintains the boundary of dorsal forerunner cell cluster during morphogenesis of the zebrafish embryonic left-right organizer

**DOI:** 10.1242/dev.132969

**Published:** 2016-07-15

**Authors:** Junfeng Zhang, Zheng Jiang, Xingfeng Liu, Anming Meng

**Affiliations:** State Key Laboratory of Membrane Biology, Tsinghua-Peking Center for Life Sciences, School of Life Sciences, Tsinghua University, Beijing 100084, China

**Keywords:** Eph, Ephrin, Left-right, Asymmetry, DFCs, Kupffer's vesicle, Embryo, Zebrafish

## Abstract

The Kupffer's vesicle (KV) is the so-called left-right organizer in teleost fishes. KV is formed from dorsal forerunner cells (DFCs) and generates asymmetrical signals for breaking symmetry of embryos. It is unclear how DFCs or KV cells are prevented from intermingling with adjacent cells. In this study, we show that the Eph receptor gene *ephb4b* is highly expressed in DFCs whereas ephrin ligand genes, including *efnb2b*, are expressed in cells next to the DFC cluster during zebrafish gastrulation. *ephb4b* knockdown or mutation and *efnb2b* knockdown cause dispersal of DFCs, a smaller KV and randomization of laterality organs. DFCs often dynamically form lamellipodium-like, bleb-like and filopodium-like membrane protrusions at the interface, which attempt to invade but are bounced back by adjacent non-DFC cells during gastrulation. Upon inhibition of Eph/ephrin signaling, however, the repulsion between DFCs and non-DFC cells is weakened or lost, allowing DFCs to migrate away. Ephb4b/Efnb2b signaling by activating RhoA activity mediates contact and repulsion between DFCs and neighboring cells during gastrulation, preventing intermingling of different cell populations. Therefore, our data uncover an important role of Eph/ephrin signaling in maintaining DFC cluster boundary and KV boundary for normal left-right asymmetrical development.

## Abstract (precis)

**Summary:** During formation of the Kupffer's vesicle (KV) – the left-right organizer in zebrafish – Eph/ephrin signaling prevents KV cells from intermingling with adjacent cells.

## INTRODUCTION

In most vertebrate species, embryos initially develop symmetrically along the prospective body midline. Following gastrulation completion, a group of epithelial cells, which may form a distinct transient organ/tissue called the left-right (LR) organizer (Blum et al., 2014), develop motile monocilia (Nonaka et al., 1998; Essner et al., 2002, 2005; Okada et al., 2005; Schweickert et al., 2007). The rotation of these cilia generates leftward fluid flow, resulting in the release of laterality cues to the left side. The laterality cues activate left-sided effector gene expression in the left lateral plate mesoderm (LPM) to regulate the formation of asymmetrical organs (Raya and Belmonte, 2006; Coutelis et al., 2014).

In zebrafish, the ciliated LR organizer for the generation of laterality cues is Kupffer's vesicle (KV), a spherical structure located in the tail region ventral to the chordal-neural hinge during early somitogenesis (Essner et al., 2002, 2005). KV progenitors are dorsal forerunner cells (DFCs) posterior to axial mesodermal progenitors at the dorsal margin and flanked by marginal mesendodermal progenitors on both sides at the onset of gastrulation (Cooper and D'Amico, 1996). During gastrulation, the axial mesodermal progenitors involute into the deep layer and migrate anteriorly, and the lateral mesendodermal progenitors also involute but this is followed by anteromedial migration. By contrast, DFCs do not involute, and instead they always stay together at the leading edge of the dorsal blastodermal margin and move as a collective mass towards the vegetal pole during gastrulation (Cooper and D'Amico, 1996; D'Amico and Cooper, 1997). An amazing phenomenon is that DFCs and their neighboring non-DFCs never intermingle though both types of cells are actively migrating. The mechanisms for maintaining the boundary between the DFC cluster and outside cells remain unknown.

Sorting of cells with distinct fates, separation of cell populations, and establishment and maintenance of tissue boundaries during embryonic development can be accomplished via different mechanisms, including differential cell adhesion, differential interfacial tension and repulsion/contact inhibition (Batlle and Wilkinson, 2012; Fagotto, 2014). Notably, the Eph/ephrin signaling system mediates cell-cell contact and repulsion (Pasquale, 2005). The Eph proteins are a large family of transmembrane receptor tyrosine kinases, and their ligands are the ephrin proteins that are also anchored to the cell surface. When an ephrin-expressing cell makes contact with an Eph-expressing cell, their extracellular domains associate to form complexes and subsequent molecular events at the interface and inside the cells alter cell membrane architecture and cell behaviors. Eph/ephrin signaling has been found to play an important role in controlling boundary formation of versatile tissues (Batlle and Wilkinson, 2012; Klein, 2012). For instance, Eph-ephrin interaction can regulate the guidance of migration of neuronal axons (Egea and Klein, 2007), the formation of eye fields (Cavodeassi et al., 2013), the formation of blood and lymphatic vessels (Mäkinen et al., 2005; Adams et al., 1999; Gerety et al., 1999; Wang et al., 1998, 2010), the separation of germ layers (Rohani et al., 2011, 2014; Fagotto et al., 2013), and the formation of somites and hindbrain segments (Durbin et al., 1998, 2000; Oates et al., 1999; Barrios et al., 2003; Cooke et al., 2005; Kemp et al., 2009). However, it is not known whether and how the ephrin-Eph interplay system could regulate the laterality of organs.

In this study, we report a crucial role of the Eph/ephrin system in embryonic asymmetrical development in the zebrafish. We found that the Eph receptor gene *ephb4b* and several ephrin ligand genes, including *efnb2b*, are expressed inside and outside the DFC cluster in a complementary fashion. Ephb4b-Efnb2b interaction provides a repulsive signal for maintaining the border between DFCs and adjacent cells, which ensures that DFCs uphold a clustering state during gastrulation cell movements to form a KV of normal size and to guarantee correct asymmetrical development subsequently.

## RESULTS

### Eph/ephrin signaling is implicated in LR development

**Fig. 1.**
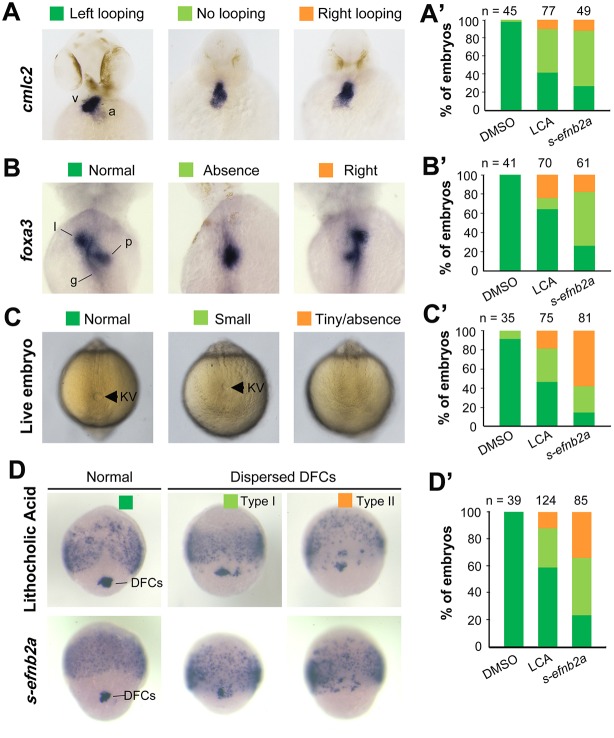
**Blockage of Eph signaling disrupts laterality development in zebrafish embryos.** Wild-type embryos treated with LCA or injected with *s-efnb2a* mRNA were analyzed. (A,A′) Defects in heart looping as visualized by *cmlc2* expression at 48 hpf. Different types of looping are shown in ventral view (A) and the proportions of embryos exhibiting each type are shown in the graph (A′). (B,B′) Defects in laterality of liver (l) and pancreas (p) as visualized by *foxa3* expression at 48 hpf, shown in dorsal view (B) with quantitative data shown in the graph (B′). (C,C′) Defects in KV at 10 SS. The tail region was viewed dorsally (C) with visible KV indicated by arrowheads. The proportions of embryos exhibiting each phenotype are shown in the graph (C′). (D,D′) Defects in DFCs aggregation as visualized by *sox17* expression at 75% ES. The representative types of embryos are shown in D with the proportions of embryos exhibiting each type shown in D′. a, atrium; g, gut/intestine; v, ventricle; n, number of embryos observed.

**Fig. 2.**
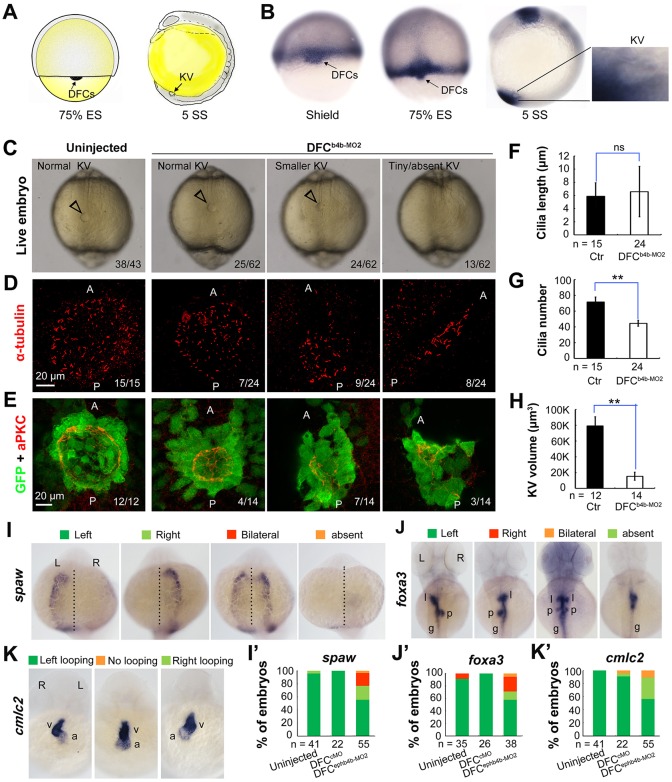
***ephb4b* is required for KV formation and LR asymmetrical development.** (A) Illustration of DFCs at 75% ES (dorsal view) and KV at 5 SS (lateral view). (B) *ephb4b* expression pattern at the indicated stages. (C-H) Effects of DFC-specific *ephb4b* knockdown on KV size (C), cilia (D,F,G) and KV lumen (E,H) as analyzed at 10 SS. KV in live embryos (C) is indicated by open arrowheads and embryo ratios with different KV sizes are indicated. Cilia were visualized by acetylated tubulin immunofluorescence (D), and cilia number (F) and length (G) were analyzed. The apical surface of KV cells in *Tg(sox17:GFP)* embryos was labeled by co-immunostaining with anti-aPKC (red) and anti-GFP antibodies (green) (E), and the KV lumen size was measured on confocal images (H). A and P indicate the anterior and posterior sites of KV, respectively. In D and E, embryo ratios with the representative pattern are indicated. Error bars indicate s.e.m.; *n*, embryo number; ns, not statistically significant; **P<0.01. (I-K′) Effect of DFC-specific knockdown on *spaw* expression at 21 SS (I; dorsal views), *foxa3* expression in liver (l), pancreas (p) and intestine (g) at 48 hpf (J; dorsal views), and *cmlc2* expression in the ventricle (v) and the atrium (a) of the heart at 48 hpf (K; ventral views). The left and right sides are indicated by L and R, respectively. The proportions of embryos exhibiting each phenotype are shown in I′-K′.

To test whether Eph/ephrin signaling is involved in LR development, we adopted two approaches to interfere with Eph/ephrin signaling in early zebrafish embryos, which was followed by examination of laterality markers, KV size and DFCs aggregation form. The first approach was to inhibit the Eph-ephrin interaction by treating embryos from 50% epiboly stage (ES) to 75% ES or bud stage with lithocholic acid (LCA), an antagonist of Eph receptors (Giorgio et al., 2011). The second approach was to block Eph receptors by injecting one-cell-stage embryos with *s-efnb2a* mRNA, which encodes the zebrafish EphrinB2a (Efnb2a) extracellular domain; this domain has binding affinity for Eph receptors and has the effect of antagonizing Eph forward signaling (Davis et al., 1994; Durbin et al., 1998). Inhibition of Eph signaling by LCA or *s-efnb2a* overexpression caused randomization of heart looping (Fig. 1A,A′) and liver position (Fig. 1B,B′) at 48 h post-fertilization (hpf), suggesting a disruption of normal laterality. We then looked into size changes of KV, the LR organizer, at the 10-somite stage (SS) when KV is well formed (Essner et al., 2005). Results showed that the majority of embryos treated with LCA or overexpressing *s-efnb2a* had a smaller or no KV (Fig. 1C,C′), indicative of a requirement of Eph/ephrin signaling for KV formation. Given that KV is formed from DFCs (as illustrated in Fig. 2A), this cluster of cells was examined by *in situ* hybridization for *sox17* expression at 75% ES. It appeared that in LCA-treated or *s-efnb2a*-overexpressing embryos some *sox17*-positive DFCs went away from the dorsal leading edge of the blastodermal margin, resulting in a reduction of DFC cell number at the normal location (Fig. 1D,D′). These results suggest that Eph/ephrin signaling is required for LR asymmetrical development probably by maintaining integrity of DFCs.

### Ephb4b in DFCs is required for KV formation and LR development

Given that LCA and *s-efnb2a* may target different Eph receptors, we set out to identify specific Eph receptors functioning in DFCs. Based on the ZFIN database (http://zfin.org), 16 Eph receptor (*eph*) genes and 12 ephrin ligand (*efn*) genes in the zebrafish genome have been annotated (Table S1). To search for Eph receptor (*Eph*) or ligand (*ephrin*/*efn*) genes expressed in DFCs, we analyzed expression patterns during midgastrulation, by whole-mount *in situ* hybridization, of nine *Eph* and seven *efn* genes that may be expressed during gastrulation (Fig. S1). *ephb4b* was found to be the only one that was highly expressed in DFCs and KV epithelia (Fig. S1A; Fig. 2B), *ephb3a* had a low level of expression in DFCs (Fig. S1A), and none of the *efn* genes examined was expressed in DFCs (Fig. S1B). The high-level expression of *ephb4b* in DFCs suggests a role in LR development.

We then decided to investigate the potential function of *ephb4b* in LR development using two translation-blocker morpholinos, ephb4b-MO1 and ephb4b-MO2 (Fig. S2A). The reporter assay revealed that ephb4b-MO2 was more effective than ephb4b-MO1 in blocking *ephb4b* expression (Fig. S2B), so this morpholino was used in subsequent experiments. Given that *ephb4b* expression during gastrulation occurs not only in DFCs but also in marginal cells (Fig. S1B), we injected ephb4b-MO2 into the yolk at the 512-cell stage, as demonstrated by others (Amack and Yost, 2004), to specifically block translation of *ephb4b 5′utr-mCherry* fusion mRNA in DFCs (Fig. S3). Compared with injection with a standard control morpholino (cMO), injection of ephb4b-MO2 into DFCs led to a smaller or tiny/absent KV (Fig. 2C), a reduced number but unchanged length of cilia (Fig. 2D,F,H), and a decreased size of the KV lumen (Fig. 2E,H) at 10 SS. Examination of the LR markers *spaw*, *foxa3* and *cmlc2* (*myl7* – Zebrafish Information Network) at later stages also revealed abnormal laterality in DFC^e^^phb4b-MO2^ embryos (Fig. 2I-K′). We found that knockdown in DFCs of *ephb4b* in wild-type (WT) embryos did not cause an increase of apoptotic cells in DFCs and knockdown in *tp53* mutant embryos still resulted in randomization of heart jogging (Fig. S4A-E), suggesting that the defects in *ephb4b* morphants are not due to increased cell death. In addition, cell proliferation within DFCs in *ephb4b* morphants was unaffected as evidenced by the comparable proportion of pH3-positive DFCs across treatment groups (Fig. S4F-J). These results indicate that Ephb4b in DFCs is essential for KV formation and organ laterality.

### Ephb4b in DFCs is crucial for maintaining the clustered state of DFCs

**Fig. 3.**
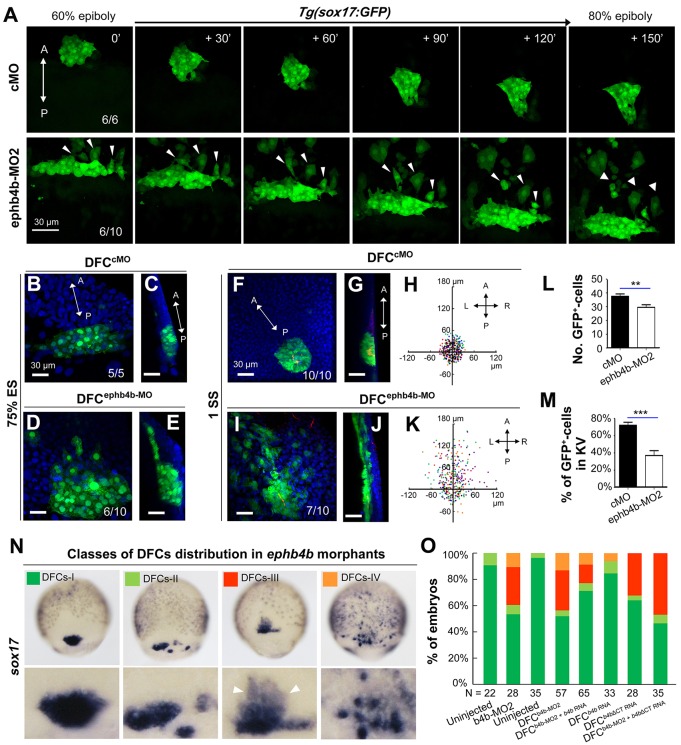
**DFC-specific *ephb4b* is required for DFC clustering.** (A) Time-lapse confocal images showing DFC migration in *Tg(sox17:GFP*) living embryos injected with MO at the 512-cell stage. The ratio of embryos with the representative pattern is indicated. Arrowheads indicate DFCs moving away from the cluster. See also Movies 1 and 2. (B-E) Aggregation of DFCs. *Tg(sox17:GFP*) embryos injected at the 512-cell stage were immunostained at 75% ES for GFP (green) and nuclei (DAPI; blue). B and D, dorsal views; C and E, lateral views. Note that some GFP-positive DFC cells have involuted to form a string of hypoblast cells in the *ephb4b* morphant (E). (F-M) Aggregation of KV cells. Injected *Tg(sox17:GFP*) embryos were immunostained at 1 SS for GFP (green), aPKC (red) and nuclei (DAPI; blue). F and I, dorsal views; G and J, lateral views. Note the presence of DFC-derived GFP-positive cells outside KV in the *ephb4b* morphant (I,J). The distribution of individual GFP-positive cells from ten embryos is illustrated by projecting them on *x*- and *y*-axes with the aPKC-labeled KV lumen as the cross-point (H,K). The number of GFP-positive cells inside and close to KV (L) and the percentage of GFP-positive KV epithelia (labeled by aPKC) (M) in ten embryos were compared. **P<0.01; ***P<0.001. (N,O) Disruption of *sox17*-expresssing DFCs in *ephb4b* morphants. The representative DFC cluster patterns are shown in N and the proportion of embryos exhibiting each pattern category are presented in O. The AP axis is indicated in A-K.

Zebrafish DFCs migrate in a collective fashion during gastrulation towards the vegetal pole (posteriorly) at the midline, whereas marginal cells flanking the DFC cluster involute and then migrate anteriorly in the hypoblast layer. We asked whether DFC migration was disrupted upon *ephb4b* depletion due to breakdown of the DFC cluster boundary. In *Tg(sox17:GFP)* transgenic embryos, which express GFP in DFCs (Chung and Stainier, 2008; Zhang et al., 2012), GFP-positive DFCs migrated posteriorly from 60% to 80% ES and DFCs were maintained always as a cohesive group (Fig. 3A; Movies 1, 2). When *ephb4b* was knocked down in DFCs, some GFP-positive cells moved away from the remaining DFC cluster. By immunofluorescence detection of GFP, we often observed that some GFP-positive DFCs in *ephb4b* morphants at 75% ES had involuted to enter the hypoblast layer (Fig. 3D,E) whereas almost all the DFCs in control embryos stayed together (Fig. 3B,C). At 1 SS, the abnormal positioning of some DFC-derived cells in *ephb4b* morphants became more obvious (Fig. 3F,G,I,J). By projecting DFC cells to a two-dimensional coordinate after immunostaining for GFP, we found that GFP-positive DFCs in control embryos were all positioned less than 60 µm away from the intersection of the midline and the marginal line (Fig. 3H), whereas GFP-positive cells in *ephb4b* morphants showed a wider dispersal pattern with some cells locating 120 µm away from the intersection (Fig. 3K). As a result, the number of aggregated GFP-positive cells (Fig. 3L) and the number of GFP-positive KV cells (labeled with aPKC) were significantly reduced (Fig. 3M). These observations suggest that *ephb4b* in DFCs is necessary for preventing individual DFCs from escaping from the cluster during vegetal/posterior migration.

Based on the distribution pattern of *sox17*-positive DFCs at midgastrulation stages, we categorized embryos into four classes: DFCs-I, a single cluster of DFCs (as seen in WT embryos); DFCs-II, several dispersed clusters of DFCs with a major one; DFCs-III, one cluster of DFCs with some DFCs moving away anteriorly following involution; DFCs-IV, many small clusters of DFCs and involuting cells (Fig. 3N). As shown in Fig. 3O, injection of ephb4b-MO2 at the one-cell stage or 512-cell stage resulted in about 50% embryos with abnormal DFC distribution. Interestingly, the majority of abnormal DFCs in *ephb4b* morphants belonged to the category DFCs-III, implying that deficiency of Ephb4b mainly allowed involution of DFCs. When full-length *ephb4b* mRNA was injected into DFCs, only 15.15% of embryos showed disruption of DFCs. Co-injection of full-length *ephb4b* mRNA and ephb4b-MO2 into DFCs gave rise to a lower proportion (23.08%) of embryos with abnormal DFCs compared with ephb4b-MO2 injection alone (49.12%), suggesting a partial rescue effect. By contrast, injection of *ephb4bΔCT* mRNA, which encodes a dominant-negative form of Ephb4b without the intracellular domain, caused involution of DFCs in 35.71% of embryos, and its co-injection with ephb4b-MO2 led to a higher proportion of similarly abnormal embryos (51.43%). This result suggests that intracellular signaling of Ephb4b is important for its function. These data collectively support the idea that *ephb4b* expression in DFCs is essential for maintaining the boundary of the DFC cluster to prevent their intermingling with neighboring cells.

The Eph gene *ephb3a* appeared to be weakly expressed in DFCs (Fig. S1A). However, knockdown of *ephb3a* in DFCs led to 16.9% of embryos (*n*=65) with disaggregated DFCs, in contrast to 13.3% of cMO-injected embryos (*n*=30) and 41.9% of ephb4b-MO2-injected embryos (*n*=62), which were done side by side (Fig. S5C,G). These results imply that *ephb3a* may play a negligible role in maintaining the clustering state of DFCs during gastrulation cell movements.

### *ephb4b* knockout mutants display DFCs and laterality defects

**Fig. 4.**
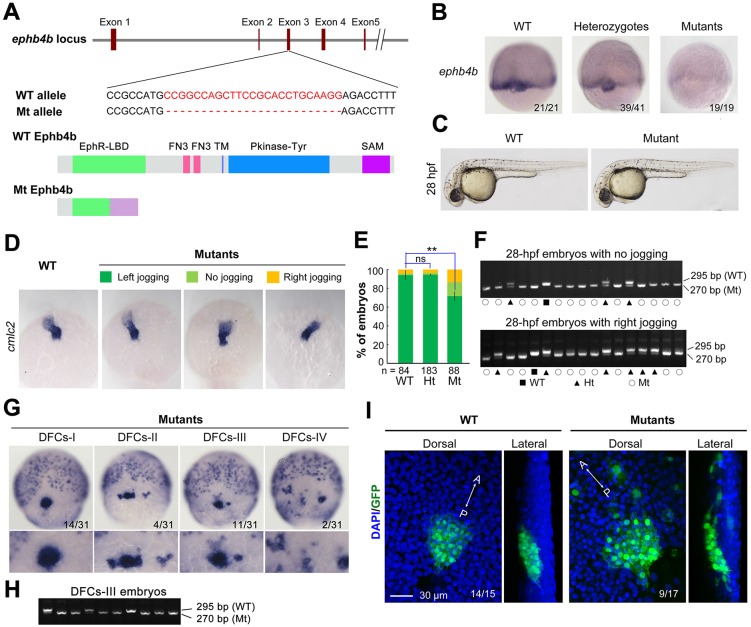
***ephb4b* genetic mutants exhibit defects in DFC aggregation and laterality.** (A) Illustration of the *ephb4b* mutant allele (Mt) generated by the Cas9 knockout approach. (B) *ephb4b* expression pattern in different types of embryos at 75% ES. The siblings were genotyped after being photographed and the proportion of embryos exhibiting the representative pattern is indicated. (C) Morphology of live WT and mutant siblings at 28 hpf. (D-F) Heart jogging was detected by probing *cmlc2* expression at 28 hpf (D) and quantified (E). ns, not significant; **P<0.01. After *in situ* hybridization, each embryo was genotyped by PCR as shown in F. (G) DFC aggregation was examined by probing *sox17* expression at 75% ES. Shown are representative DFC patterns in mutants only. The DFC region is enlarged in the lower panels. (H) Representative genotyping results for DFCs-III embryos. (I) Embryos at the bud stage were immunostained for GFP expression and observed by confocal microscopy. The AP axis is specified.

To substantiate further the requirement of *ephb4b* for DFC aggregation and organ laterality, we generated a genetic mutant line of *ephb4b* using clustered regularly interspaced short palindromic repeats (CRISPR)/Cas9 technology in *Tg(sox17:GFP)* fish. The mutant allele carries a 25-bp deletion in the third exon, resulting in a premature stop codon and presumably a truncated peptide lacking the transmembrane and other C-terminal domains (Fig. 4A). The amount of *ephb4b* transcripts was slightly reduced in heterozygotes and almost eliminated in homozygous mutants at 75% ES (Fig. 4B). Although the homozygous mutants, which were confirmed by genotyping (Fig. 4F), looked morphologically normal at 28 hpf (Fig. 4C), 28.4% (*n*=88) of mutants had no or right jogging of the heart in contrast to 6% (*n*=84) and 5.5% (*n*=183) of WT and heterozygous siblings, respectively (Fig. 4D,E). *In situ* hybridization results revealed that 54.5% of mutants (*n*=31) showed variable degrees of DFC disaggregation at 75% ES (Fig. 4G,H), whereas DFC disaggregation occurred in only 8.3% (*n*=36) and 12.5% (*n*=4) of WT and heterozygous siblings, respectively. The escape and involution of some DFCs were also detected in mutants at the bud stage (Fig. 4I). *In vivo* dynamic observations showed that no DFCs in WT sibs (*n*=14) involuted or escaped (Fig. S6A; Movie 3) whereas DFCs beneath axial mesodermal precursors in mutants (*n*=13) involuted often (Fig. S6B; Movie 3). Based on single focal, multi-focal and 3D reconstituted movies (Movie 4; Fig. S6C), we found that two adjacent DFCs (d1 and d2) in mutant embryos could be separated by insertion of a mesoderm cell (m1). Taken together, these results indicate that *ephb4b* knockout mutants have defects in DFC aggregation and laterality, essentially phenocopying *ephb4b* morphants.

### *efnb2b* regulates DFC aggregation, KV formation and laterality

**Fig. 5.**
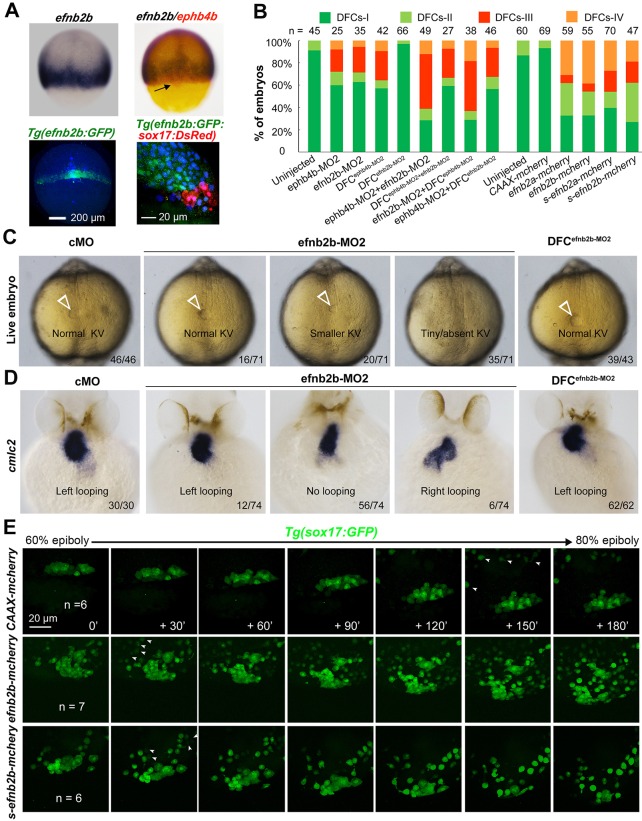
***efnb2b* expression outside DFCs is required for DFC aggregation, KV formation and laterality development.** (A) *efnb2b* is not expressed in DFCs. Top panels: *efnb2b* (black)/*ephb4b* (red) *in situ* patterns with DFCs indicated (arrow) at 75% ES; bottom panels: *Tg(efnb2b:GFP)* embryo expressing GFP in the blastodermal margin at shield stage (left panel; dorsal view), and GFP^+^ mesoderm precursors located next to DsRed^+^ DFCs in *Tg(efnb2b:GFP;sox17:DsRed)* embryo at 75% ES (right panel: lateral view). (B) *efnb2b* knockdown in the whole embryo caused disaggregation of DFCs. Wild-type embryos were injected at the one-cell or 512-cell stage (DFC^X^) and probed at midgastrulation stages for *sox17* expression by *in situ* hybridization. The distribution patterns of DFCs were categorized as shown in Fig. 3N. (C,D) Effect of *efnb2b* knockdown on KV at 10 SS (C) and on *cmlc2*-labeled heart looping at 48 hpf (D). Arrowheads indicate the KV. Proportion of embryos exhibiting each phenotype is indicated. (E) Time-lapse observation of DFC migration during gastrulation in living embryos. *Tg(sox17:GFP)* embryos injected with the indicated mRNA at the one-cell stage were dynamically observed by confocal microscopy starting at 60% ES. Arrowheads indicate GFP-positive endoderm progenitors. The relative time points are indicated. Embryos were orientated with anterior to the top. *n*, number of observed embryos. See also Movies 5-7.

We noted that several of the *efn* genes examined, including *efnb2a*, *efnb2b* and *efnb3b*, and probably also *efna5a* and *efna5b*, were expressed in axial mesodermal and lateral blastodermal marginal cells adjacent to the DFC cluster but not in DFCs (Fig. S1B). We chose *efnb2b* for deep study because of its high expression levels. Double *in situ* analysis of *efnb2b* and *ephb4b* confirmed that *efnb2b* was not expressed in DFCs (Fig. 5A, top panel). In *Tg(efnb2b:GFP;sox17:DsRed)* transgenic embryos, *efnb2b*:GFP^+^ cells were found immediately adjacent to DsRed^+^ DFCs in the dorsal margin at 75% ES (Fig. 5A, bottom panel). To knock down *efnb2b* expression, efnb2b-MO1 and efnb2b-MO2 were used (Fig. S7A). The reporter assay revealed that both morpholinos were effective but efnb2b-MO2 was more effective (Fig. S7B-D). Injection of efnb2b-MO2 at the one-cell stage caused dispersal and involution of DFCs at midgastrulation stages (Fig. 5B; Fig. S5F), a smaller or unrecognizable KV at 10 SS (Fig. 5C) and randomized heart looping at 48 hpf (Fig. 5D). However, injection of efnb2b-MO2 at the 512-cell stage disrupted neither DFC clustering and KV formation nor heart laterality (Fig. 5B-D). Although co-knockdown of *efnb2b* and *ephb4b* in the whole embryo decreased DFC abnormalities, co-knockdown in DFCs resulted in DFC abnormalities at degrees comparable to *ephb4b* DFC knockdown alone. These results indicate that, unlike *ephb4b*, which functions cell-autonomously, *efnb2b* regulates DFC aggregation, KV formation and laterality in a non-cell-autonomous fashion.

**Fig. 6.**
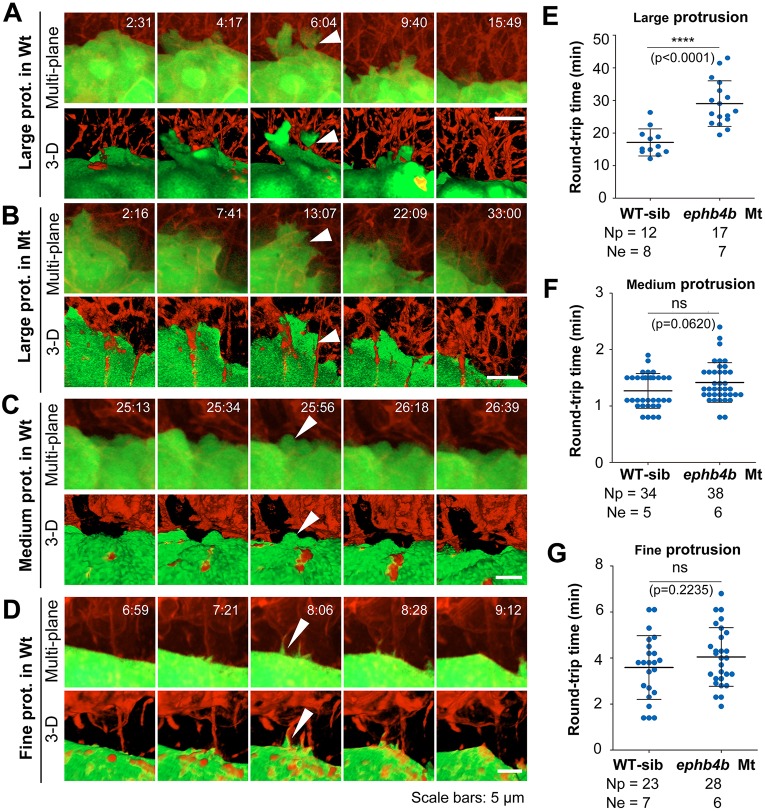
**Contact duration between DFCs and non-DFC cells is increased by deficiency of Eph/ephrin signaling.** The membrane of embryonic cells from crosses of *ephb4b^+/−^;Tg(sox17:GFP)* fish was labeled by expressing exogenous CAAX-mCherry (red) and embryos were observed during midgastrulation. (A-D) Multi-focal and 3D reconstituted time-lapse images of different types of protrusions of DFCs in *ephb4b* mutant (Mt) (B) and WT siblings (A,C,D). The full process of a single protrusion is shown with time points indicated. The arrowheads indicate protrusions at the middle phase. See also Movies 8 and 9. (E-G) Round-trip time of protrusions. The start and end time points of the process for each protrusion were judged from single-focal, multi-focal and 3D movies. Only well-recognized protrusions were included. Np, number of protrusions; Ne, number of embryos.

We next investigated the effect of *efnb2b* overexpression on DFC aggregation by injecting *efnb2b-mCherry* or *s-efnb2b*-*mCherry* (encoding only the Efnb2b extracellular domain) mRNA at the one-cell stage. Results showed that both of these mRNA species caused dispersal and involution of DFCs in a large proportion of injected WT embryos (Fig. 5B). Time-lapse recording also confirmed that DFCs in injected *Tg(sox17:GFP)* embryos moved away from the original cluster during gastrulation (Fig. 6E; Movies 5-7). These results collectively suggest that either ectopic activation or inhibition of Eph/ephrin signaling in the whole embryo including DFCs gives rise to loss of DFC integrity.

### Eph/ephrin signaling stimulates cell-cell contact and repulsion

To understand why abnormal Eph/ephrin signaling disaggregated DFCs, we observed the dynamics of the interface between DFCs and adjacent non-DFC cells during gastrulation. We found that DFCs immediately next to axial mesodermal precursors (AMs) often formed protrusions towards the AMs and retracted or were pushed back immediately after touching the membrane of the AMs (Fig. 6A-D; Movies 8, 9). We categorized DFC protrusions into three types, lamellipodium-like large, bleb-like medium and filopodium-like fine, and counted the ‘round-trip’ time of each recognizable protrusion. The round-trip time for all types of protrusions in *ephb4b* mutants appeared to be longer than in WT siblings although the difference was statistically significant only for large protrusions (Fig. 6E-G). We hypothesize that deficiency of Eph/ephrin signaling due to loss of Ephb4b on the membrane of DFCs causes a decrease of repulsion force and an increase of cell adhesion between DFCs and non-DFC cells, consequently allowing involution and escape of DFCs.

**Fig. 7.**
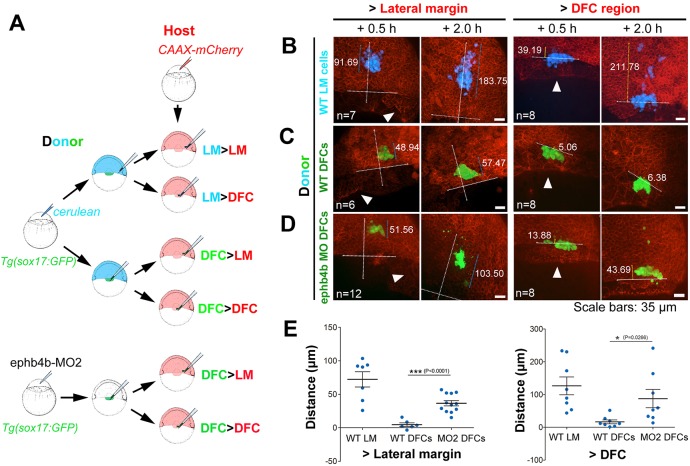
**Migration behavior of transplanted DFCs and lateral mesodermal precursors.** (A) Illustration of six types of transplantation. (B-D) Confocal images showing distribution of transplanted cells in host embryos at two time points post-transplantation. The horizontal line indicates the leading edge of the blastodermal margin; the long vertical line indicates the animal pole (top) to vegetal pole (AV) axis; the shorter line indicates the distribution distance (µm). Arrowheads indicate the presumed DFC cluster. See also Movie 10 for examples. (E) Distribution distance of transplanted cells 2 h post-transplantation. For transplantation to lateral margin, the distance was the scattering distance of transplanted cells along the AV axis; for transplantation to the DFC region (vegetal to dorsal blastodermal margin), the distance was measured from the cell most close to the animal pole to the blastodermal margin.

We next investigated DFCs behaviors in different regions of embryos by performing six types of transplantation (Fig. 7A). Lateral marginal (LM) cells transplanted to either the LM or DFC region of host embryos involuted and migrated towards the animal pole (Fig. 7B,E; see an example in the left panel of Movie 10), indicating a lack of intrinsic mechanisms and extrinsic pressures to aggregate LM cells. WT DFCs transplanted to the LM or DFC region remained as a cluster as if they did not migrate in any directions over the observed period (1.5 h) (Fig. 7C; Movie 10, middle panel). However, DFCs depleted of *ephb4b*, when transplanted to the LM, apparently migrated a considerably long distance towards the animal pole (Fig. 7D, left panel; Movie 10, right panel; Fig. 7E). These results suggest that transplanted DFCs possess a self-clustering activity to prevent intermingling with surrounding lateral mesodermal cells and this activity vanishes with loss of Ephb4b. Interestingly, transplanted DFCs depleted of *ephb4b* kept clustering in the host DFC region (Fig. 7D, right panel), raising one possibility that the transplanted *ephb4b*-deficient DFCs might not directly contact axial mesodermal precursors due to the presence of normal host DFCs. However, this result indicates that Ephb4b expressed in DFCs may not be required for cell adhesion between DFCs.

In vertebrate embryos, interaction between ephrin ligands and Eph receptors shows a certain degree of selectivity (Pasquale, 2004; Rohani et al., 2014). To test repulsion activity stimulated by Ephb4b-Efnb2b interaction, we mixed *ephb4b*-overexpressing and *efnb2b*-overexpressing embryonic cells, which were differentially labeled, and observed cell behavior in a hanging drop assay (Fig. S8A). Results indicated that *efnb2b*-overexpressing cells formed aggregates within the hanging drop, separate from the *ephb4b*-overexpressing cells (Fig. S8C-E). Thus, forced differential expression of *ephb4b* and *efnb2b* is sufficient to separate different cell populations.

**Fig. 8.**
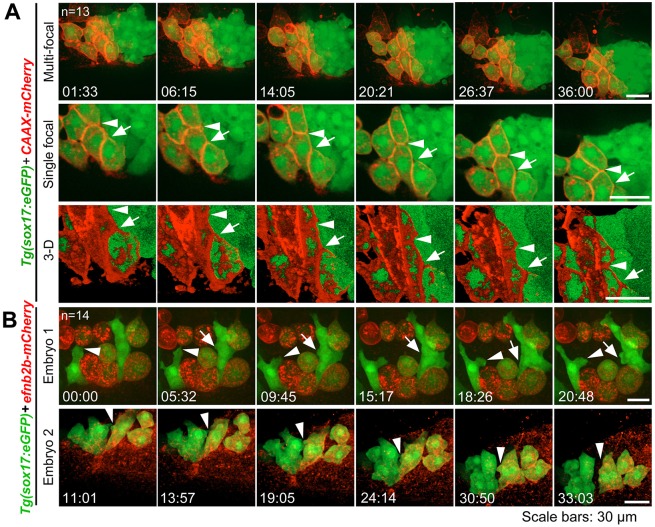
**DFCs ectopically expressing *efnb2b* fail to aggregate with normal DFCs.** *CAAX-mCherry* or *efnb2b-mCherry* mRNA was injected into the dorsal yolk of *Tg(sox17:GFP*) embryos at the 128- to 256-cell stages and embryos with uneven expression of the foreign gene in DFCs during early gastrulation were chosen for observation. (A) CAAX-mCherry-positive DFCs stayed together with other DFCS. (B) Efnb2b-mCherry-positive DFCs disaggregated with other DFCs. Shown are two representative embryos that expressed different levels of Efnb2b-mCherry. All embryos are dorsally viewed with arrows and arrowheads indicating boundaries (A) or protrusions (B). Time points are indicated. See also Movies 11 and 12.

We further tested the role of Eph/ephrin signaling in separating DFCs from non-DFC cells by overexpressing *mCherry* (control) or *efnb2b-mCherry* in a subset of DFCs via early blastula injection. Results showed that mCherry-expressing DFCs kept clustered with adjacent mCherry-negative DFCs during gastrulation although they sent out protrusions occasionally in between the latter (Fig. 8A; Movie 11). By contrast, Efnb2b-mCherry-positive DFCs were unable to aggregate stably with Efnb2b-mCherry-negative DFCs (Fig. 8B; Movie 12). Careful observation disclosed that *efnb2b*-negative DFCs actively extended membrane protrusions to neighboring *efnb2b*-positive DFCs and that the protrusions were bounced back when they touched the membrane of the latter (Fig. 8B; Movie 12). Thus, artificial introduction of Ephb4b/Efnb2b signaling in between DFCs can cause disaggregation of DFCs, supporting the idea that this signaling mediates cell-cell contact and repulsion.

### Eph/ephrin-activated RhoA is important for DFC aggregation

**Fig. 9.**
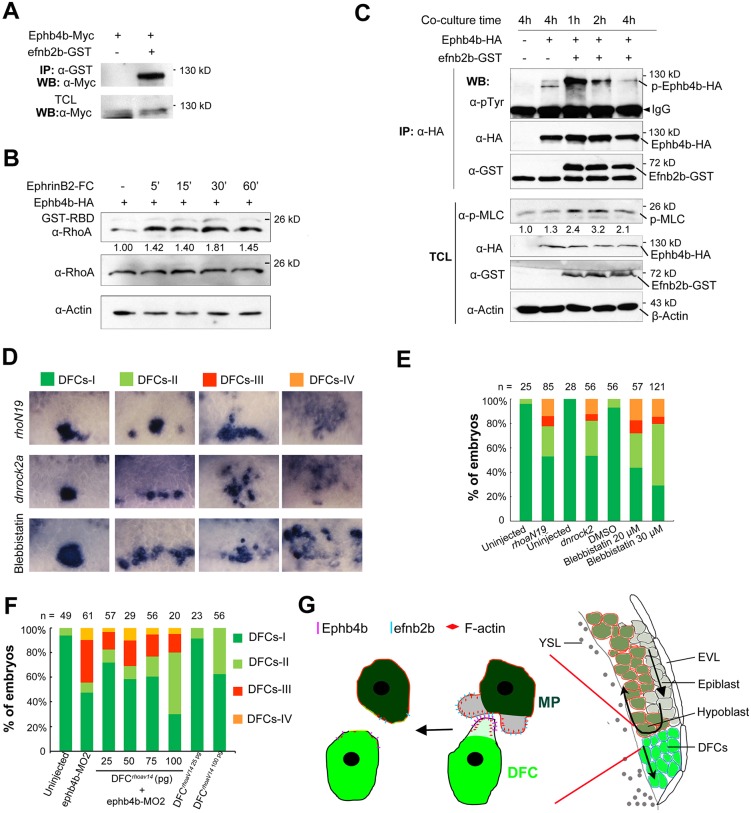
**Ephb4b/Efnb2b signaling regulates DFC clustering through the RhoA pathway.** (A) Ephb4b physically associated with Efnb2b. *Myc*-tagged zebrafish *ephb4b* and *GST*-tagged zebrafish *efnb2b* were transfected into HEK293T cells. (B) Ephb4b forward signaling activated RhoA signaling. *Ephb4b-Flag* and *GST-rhotekin-RBD* were transfected into HEK293T cells. The relative ratio of active RhoA is indicated. (C) Increase of p-MLC level by Ephb4b-Efnb2b interaction. Ephb4b-HA-expressing and Efnb2b-GST-expressing HEK293T cells were mixed and co-cultured, followed by immunoprecipitation and western blotting. The relative ratio of p-MLC is indicated. (D,E) Inhibition of the RhoA signaling pathway or actomyosin activity disaggregated DFCs. Embryos at the 512-cell stage were injected with *rhoaN19* or *dnrock2a* mRNA or treated from 50% to 65% ES with blebbistatin, followed by probing *sox17* expression at 65% ES. Representative DFC patterns are shown in dorsal view (D) with quantification in E. (F) Rescue of ephb4b-MO2-induced DFC dispersal by constitutively active RhoA (RhoaV14). One-cell-stage embryos were injected with ephb4b-MO2 alone, or re-injected at the 512-cell stage with different doses of *rhoaV14* mRNA, and examined for *sox17* expression at 75% ES, The ratios different classes (as shown in D) were calculated. (G) Model of Ephb4b/Efnb2b signaling regulation of DFC aggregation. EVL, envelope layer; MPs, mesodermal progenitors; YSL, yolk syncytial layer. See the Discussion for explanation.

Eph receptors bound by ephrin ligands can activate downstream effectors, such as Rho GTPases, in the cytoplasm to regulate cytoskeletal dynamics (Lisabeth et al., 2013). Co-immunoprecipitation analysis indicated that zebrafish Efnb2b associated with zebrafish Ephb4b when co-transfected into HEK293T cells (Fig. 9A). In HEK293T cells transfected with zebrafish *ephb4b*, the level of active RhoA (GTP-RhoA) was increased by treatment with mouse ephrin-B2-Fc (Fig. 9B). Furthermore, *efnb2b*- Ephb4b interaction stimulated Ephb4b and Myosin Light Chain 2 (MLC2) phosphorylation (Fig. 9C), a downstream effector of RhoA/Rock2 signaling. In cMO-injected embryos at 75% ES, active RhoA and p-MLC2 were enriched at the DFC boundary, but were absent in *ephb4b* morphants (Fig. S9). These data collectively indicate that Ephb4b/Efnb2b interaction could activate downstream RhoA/Rock2 signaling at the boundary of DFCs.

Finally, the effect of Rho GTPases on DFC aggregation was investigated by overexpressing *rhoaN19* mRNA or *dnrock2a* mRNA, which encode a dominant-negative form of zebrafish RhoA or Rock2a, respectively. Overexpression of either *rhoaN19* or *dnrock2a* led to scattering of DFCs (Fig. 9D,E), which mimicked *ephb4b* morphants (Fig. 3N,O) or *ephb4b* mutants (Fig. 4G). Overexpression of *rhoaN19* within DFCs led to no heart jogging (24/43) or right heart jogging (5/43) at 28 hpf, which was in contrast to 100% of left heart jogging in the same batch of uninjected embryos (*n*=29). When cytokinesis was interrupted by treating embryos during gastrulation with blebbistatin, a myosin II inhibitor (Straight et al., 2003; Limouze et al., 2004), DFCs also failed to stay together (Fig. 9D,E). DFC-specific injection of *rhoaV14* mRNA, which encodes a constitutively active RhoA, also disrupted clustering of DFCs in a dose-dependent fashion; but, importantly, low dose (25 pg) *rhoaV14* mRNA injection was able to compromise DFC scattering in *ephb4b* morphants (Fig. 9F). Therefore, we propose that the function of Ephb4b forward signaling in DFC boundary maintenance is exerted, at least in part, by downstream Rho GTPases.

## DISCUSSION

The ciliated embryonic organ of asymmetry is a transient tissue, which generates laterality cues mainly by fluid flow for asymmetrical development. It is unclear how the progenitors of this transient tissue are prevented from mixing with other types of cells during embryonic development. In this study, using the zebrafish model system, we demonstrate that Eph receptors and ephrin ligands are complementarily expressed in DFCs and adjacent non-DFC cells. Eph/ephrin signaling mediates cell-cell contact-dependent repulsion between DFCs and their neighboring cells and thus helps maintain their boundary, which essentially allows the formation of a KV of normal size.

Fig. 9G illustrates a model of how Ephb4b/Efnb2b signaling might regulate DFC aggregation. DFCs (green) at the leading edge of the blastodermal margin express *ephb4b* and migrate vegetally/posteriorly. The presumptive mesodermal progenitors (dark green) anterior to the DFCs express *efnb2b* (and also other *efn* genes), which involute into the deep layer and then migrate anteriorly. A DFC and a mesodermal progenitor form membrane protrusions towards each other; once touching the membrane of the opposite cell, Eph/ephrin-mediated contact and repulsion cause retraction of the protrusions, which may result in dynamic changes of actin filament and alteration of local membrane characteristics.

DFCs and their neighboring mesoderm precursors both form membrane protrusions at the boundary, mainly bleb-like and sometimes filopodia- and lamellipodia-like, which are characteristic of migrating embryonic cells (Trinkaus, 1973; Diz-Muñoz et al., 2010; Ridley, 2011), and these protrusions retract immediately upon touching the surface of the opposite cell (Fig. 6; Movie 8) so that different types of cells are prevented from intermingling. When Eph/ephrin signaling is blocked, mesodermal precursors are able to insert between and form stable contact with DFC cells, which causes separation of adjacent DFCs and collective migration of dispersed DFCs with the mesodermal precursors (Fig. S6B,C). The maintenance of the boundary between DFCs and neighboring cells during gastrulation relies on differentially expressed Eph receptors and ephrin ligands and Eph/ephrin signaling-induced cell detachment, which are similar to processes that occur during *Xenopus* germ layer separation (Rohani et al., 2011, 2014; Fagotto et al., 2013). Usually, Eph/ephrin-mediated cell detachment and repulsion involves endocytosis of Eph-ephrin complexes, proteolytic cleavage of proteins, cytoskeleton and extracellular matrix reconstitution, and so on (Zimmer et al., 2003; Pitulescu and Adams, 2010; Solanas et al., 2011; Janes et al., 2005; Lisabeth et al., 2013). It remains to be determined which molecules are involved and how they regulate those follow-up events during the collective migration of DFCs.

Impairment of Ephb4b/Efnb2b leads to disaggregation of DFCs, suggesting a possible involvement of cell adhesion in DFCs clustering. We could not detect Cdh1 alteration in DFCs of *ephb4b* morphants or mutants (data not shown). The fact that transplanted *ephb4b*-deficient DFCs remain aggregated in the host DFC region (Fig. 7D, right panel) implies that *ephb4b* may not be required for cell adhesion between DFCs. Besides, although *cdh1* knockdown or overexpression also caused disaggregation of DFCs, either manipulation could not reverse the effect of *ephb4b* knockdown on DFCs aggregation (data not shown), suggesting that *ephb4b* is unlikely to regulate Cdh1 within DFCs.

Different Eph receptors selectively associate with ephrin ligands (Pasquale, 2004). Among our tested set of *eph*/*efn* genes, *ephb4b* is the only Eph member gene highly expressed in DFCs and *efnb2b* is a ligand gene highly expressed in mesodermal precursors adjacent to DFCs. We demonstrate that Ephb4b can associate with *efnb2b* and activate the downstream RhoA pathway (Fig. 9A-D). It appears that other *efn* genes are also expressed in the blastodermal margin (Fig. S1B). It remains to be determined whether those ephrin products interact with Ephb4b and play a role in DFC clustering.

Other vertebrate species may also form a ciliated LR organizer during late gastrulation or early neurulation, e.g. the node in mouse (Essner et al., 2002; Lee and Anderson, 2008), gastrocoel roof plate in *Xenopus* (Essner et al., 2002; Schweickert et al., 2007), and the posterior notochordal plate in rabbit (Feistel and Blum, 2006; Blum et al., 2007). Currently, it is not known how the progenitors of the LR organizer in other species are prevented from intermingling with bordering cells. We note a report that 17% of *Ephb4* knockout embryos at embryonic day 8.75-9.0 exhibit incomplete heart looping (Gerety et al., 1999), which suggests a potential role of Ephb4 signaling in mouse laterality development. Nevertheless, future efforts need to test the conservative role of Eph/ephrin signaling in the formation of the LR organizer and laterality development.

## MATERIALS AND METHODS

### Zebrafish strains and drug treatment

Wild-type Tuebingen zebrafish, *Tg(sox17:GFP)^s870^* transgenic line (Chung and Stainier, 2008) and *tp53^M214K^* mutant line (Berghmans et al., 2005) were used with ethical approval from the Animal Care and Use Committee of Tsinghua University. *Tg(efnb2b:GFP)* (Liu et al., 2011) and *Tg(sox17:DsRed)* lines were established from embryos injected with transgenic constructs. For each experiment, the same batch of embryos was used. For drug treatment, embryos incubated in Holtfreter solution were dechorionated at 50% ES, transferred to Holtfreter solution containing 200 µM LCA or 20 µM (-)-blebbistatin (Cayman Chemical), and raised until 75% ES (for probing *sox17*) or bud (for other assays) stages.

### Generation of *ephb4b* mutant line using the CRISPR/Cas9 approach

Two gRNAs were designed to target the sequences 5′-GGAGATCCGCTTCACCATGATGG-3′ and 5′-CCATGCCGGCCAGCTTCCGCACC-3′ within the third exon of the *ephb4b* locus. A mixture of 50 pg of each of the gRNAs was co-injected with 300 pg *Cas9* mRNA into one-cell-stage embryos. The mutant alleles were identified by PCR using the primers 5′-TGTGTCAGTGGGAGGAGATGAGC-3′ and 5′-GTACCTTGCTGTAGGGGTTCTC-3′, followed by sequencing. Only one mutant line that carries a 25-bp deletion within the third exon was kept.

### Expression vectors

For mRNA synthesis or cell transfection, the corresponding cDNA sequence was inserted into mCherry-tagged pXT7, Flag-tagged pCS2, Myc-tagged pCMV5 or GST-tagged PEBG1 vector. *dnrock2a* was used as previously described (Zhang et al., 2009). All mRNAs were synthesized using the mMessage mMachine T7/SP6 kit (Ambion).

### Morpholinos and mRNA injection

The antisense morpholino oligonucleotides (MOs) were synthesized by Gene Tools, LLC. The sequences for MOs were as follows: ephb4b-MO1, 5′-GATCACTTCAGTCTCTCTGATATGA-3′; ephb4b-MO2, 5′-AATCCAGCAAACACGATCCATCTCA-3′; ephb4b-sMO, 5′-TCATCTCCTCCCACTGACACAACAC-3′; efnb2b-MO1, 5′-CGCAGGCACTAAAACGAGCACAATC-3′; efnb2b-MO2, 5′-TAACTCCAGACAGTCGCATCCATTG-3′; cMO, 5′-CCTCTTACCTCAGTTACAATTTATA-3′; ephb3a-MO, 5′-GACAGAAAGTCTCGTTAAATCTCAG-3′; efnb2a-MO, 5′-CGGTCAAATTCCGTTTCGCGGGA-3′ (Cooke et al., 2005).

For knockdown or overexpression in the whole embryo, reagents were injected at the one-cell stage. For knockdown or overexpression specifically in DFCs, reagents were injected together with 1 µg/µl Rhodamine into the yolk cell at around the 512-cell stage, followed by selection of Rhodamine-positive embryos at 60%-70% ES.

Unless otherwise stated, the injection doses were as follows: 10 ng ephb4b-MO2, 7 ng efnb2b-MO2, 300 pg *ephb4b* or *ephb4b-mCherry*, 400 pg *ephb4bΔCT-mCherry*, 250 pg *CAAX-mCherry*, 250 pg *s-efnb2a-mCherry*, 250 pg *s-efnb2b-mCherry*, 250 pg *efnb2a-mCherry*, 250 pg *efnb2b-mCherry*, 25 pg *rhoaV14*, 300 pg *rhoaN19* and 300 pg *dnrock2a* mRNA.

### Whole-mount RNA *in situ* hybridization

For *eph* and *efn* genes, a specific sequence was amplified with a specific pair of primers with the T7 promoter sequence in the lower primer and used for *in vitro* RNA synthesis as described (Thisse and Thisse, 2008). Other probes were described previously (Zhang et al., 2012; Liu et al., 2013). Synthesis of digoxigenin- or fluorescein-labeled antisense RNA probes and whole-mount *in situ* hybridization were performed as described previously (Zhang et al., 2012; Thisse and Thisse, 2008).

### Whole-mount immunofluorescence assay

Whole-mount immunofluorescence assay was performed essentially as described previously (Zhang et al., 2012; Xue et al., 2014). Primary antibodies for immunofluorescence were: mouse anti-GFP (1:200; sc-9996, Santa Cruz), rabbit anti-GFP (1:800; Ab290, Abcam), mouse anti-acetylated tubulin (1:400; T6793, Sigma), rabbit anti-aPKC (1:100; sc-216, Santa Cruz), rabbit anti-pH3 (1:200; #9701, Cell Signaling Technology), rabbit anti-active capase3 (1:200; BD 559565, BD Biosciences), mouse anti-GST (1:400; BE2075, EASYBIO), rabbit anti-p-MLC2 (Thr18/Ser19) (1:200; #3674, Cell Signaling Technology), and rabbit anti-RhoA (1:500; BS1782, Bioworld) antibodies. Secondary antibodies were Alexa Fluor 488- or 649-conjugated anti-mouse IgG (1:100; 115-545-003 and 115-605-003, Invitrogen) and Alexa Fluor 488- or 649-conjugated anti-rabbit IgG (1:100; A11008 and A27040, Invitrogen) antibodies. For imaging, the embryo region containing DFCs or KV was dissected, embedded in the mounting medium and were observed under a Zeiss LSM META confocal microscope. Confocal images of DFCs were acquired as a *z*-series with a step-size of 2 μm and those of KV as a *z*-series with a step-size of 1 μm.

### Live imaging

To visualize DFC migration, embryos at a desired stage were manually dechorionated and mounted in 1% low-melting-point agarose. Time-lapse multiple-focal-plane (4D) microscopy was then performed at 25°C under an Olympus FV1000 multi-photon system using a Plan Apochromat 40×/1 W dipping objective or at 28°C with a Perkin Elmer Spinning Disk confocal microscope system using 20×/40×/60× (Silicon Oil) objectives. Movies were processed using Imaris software 7.1.0 (Bitplane AG) or Image-Pro Plus 6.0 software (Media Cybernetics) or Volocity 6.1.1 (Perkin Elmer). 3D reconstructions were processed with Volocity 6.1.1 (Perkin Elmer). Embryos were individually genotyped after observation when necessary.

### Transplantation assay

*Tg (sox17:GFP)* transgenic embryos were injected with 250 pg *cerulean* mRNA to label donor cells or with ephb4b-MO2 at the one-cell stage. WT host embryos were injected with 260 pg *CAAX-mRNA* at the one-cell stage to label the plasma membrane. Donor DFCs or lateral marginal cells were transplanted to lateral margin of host embryos at about 60% ES or to the DFC region vegetal to the dorsal margin. About 0.5 h post-transplantation, host embryos were mounted in 1% low-melting-point agarose and observed for 1.5 h with a Perkin Elmer Spinning Disk confocal microscope using a 20× objective at 28°C.

### Immunoprecipitation and western blot

Western blotting and co-immunoprecipitation were performed as previously described (Liu et al., 2013). GST-EfnB2b fusion protein expressed in HEK293T cells was enriched using Glutathione Sepharose 4B beads (GE Healthcare). After washing with cell lysis buffer, the beads were incubated with the lysates of Ephb4b-Myc-transfected HEK293T cells for 2 h at 4°C, followed by washing with cell lysis buffer again. The final eluent was analyzed by western blotting. The antibodies used were: mouse anti-Myc (1:5000; sc-40, Santa Cruz), mouse anti-Flag (1:1000; M185, MBL), rabbit anti-p-MLC2 (1:1000; #3674, Cell Signaling Technology), mouse anti-GST (1:5000, EASYBIO) and goat anti-zebrafish Ephrin-B2 (1:1000; AF1088, R&D Systems).

### RhoA activity assay

Mouse Ephrin-B2-Fc (50598, Sino Biological) or control human Fc (2 μg/ml; 10702-HNAH, Sino Biological), which was pre-clustered by goat anti-human IgG antibody (SSA016, Sino Biological), was applied to *ephb4b-Flag/ GST-rhotekin-RBD-*transfected HEK293T cells and cells were incubated for different durations. After treatment, cells were lysed and the lysate was precipitated using GST beads for 1.5 h. After precipitation, the beads were washed twice with lysis buffer. The final eluent was analyzed by western blotting using anti-RhoA antibody (1:500; #2117, Cell Signaling Technology).
